# Virginia State Dental Association

**Published:** 1870-12

**Authors:** Geo. F. Keesee

**Affiliations:** Secretary.


### ARTICLE IV.

### *Virginia State Dental Association.*

RICHMOND, Nov. 18th, 1870.

PROF. F. J. S. GORGAS,

*Editor American Journal of Dental Science.*

DEAR SIR:—In obedience to the notice you kindly gave in the October No. of your most excellent Journal, quite a goodly number of the dentists of our State met in Richmond on Thursday November 3d, 1870, for the purpose of organizing a State Dental Association. The spacious rooms of Dr. Judson B. Wood had been kindly offered for the place of meeting. At the hour appointed the meeting was called to order by Dr. Hugh M. Grant, of Abingdon, who nominated Dr. Jas. F. Thompson of Fredericksburg, as Chairman, and requested the writer to act as Secretary. Dr. Thompson in a very neat speech thanked those present for the honor conferred upon him, and explained the object of the meeting to be the organization of an Association having in view the cultivation of the science and art of dentistry and all its collateral branches, to elevate and sustain the professional character of dentists and to promote amongst them mutual improvement, social intercourse and good will.

On motion the chairman appointed a committee of five, consisting of Drs. H. M. Grant, J. B. Wood, T. M. Henley, J. W. Scribner and G. B. Steel to prepare business for the meeting.

During the absence of the committee, the chairman read letters from several of the most eminent members of the profession in the State, regretting that business and other previous engagements prevented their attendance, but assuring that their influence should ever be exerted for the furtherance of the contemplated object, and urging that all should use their best endeavors to push forward so noble a cause.

The committee on returning reported a Constitution and By-Laws for the government of the Association, which in all honor to them I must say compares favorably with those which govern any Dental Association throughout the country. The Association was then organized under the name and title of the Virginia State Dental Association, and adopted the Constitution and By-Laws offered by the committee and at once proceeded to the election of officers, which resulted in the election of

Dr. Jas. F. Thompson of Fredericksburg, as President; Dr. Hugh M. Grant of Abingdon, 1st Vice President; Dr. Wm. M. Dorset, of Ballsville, 2d Vice President; Dr. T. M. Henley of King and Queen Co., 3d Vice President; Dr. Geo. F. Keesee of Richmond, Secretary, Dr. Judson B. Wood of Richmond, Treasurer; Drs. Hugh M. Grant, Jas. A. Chapman, and F. A. Jeter, as Executive Committee.

After the completion of all business, at a late hour the the Association adjourned to meet in Richmond on the first Thursday in November, of the next year, (1871,) the members returning to their homes enthused and feeling in their heart of hearts that a brighter era was beginning to dawn upon the dental profession in our State, and that the time was speedily coming when the days of "quack's" shall have been numbered. We earnestly hope at our next meeting to find a much larger number of the dentists of the State assembled; the old veterans coming to give their experience to the younger practitioners, and the latter coming with ambition and zeal to push forward the good work which has been commenced in our midst.

Very Truly Yours,

Geo. F. Keesee, D. D. S., Secretary.

---

